# Autonomic Nervous System and Cognitive Impairment in Older Patients: Evidence From Long-Term Heart Rate Variability in Real-Life Setting

**DOI:** 10.3389/fnagi.2020.00040

**Published:** 2020-03-11

**Authors:** Anna Maria Dalise, Raffaele Prestano, Renata Fasano, Antonio Gambardella, Michelangela Barbieri, Maria Rosaria Rizzo

**Affiliations:** Department of Advanced Medical and Surgical Sciences, University of Campania “Luigi Vanvitelli”, Naples, Italy

**Keywords:** heart rate variability, cognitive impairment, autonomic nervous system, aging, elderly patients

## Abstract

**Background**: In geriatric age, cognitive impairment and cardiovascular disorders are frequent comorbidities. Age-related anatomical and functional cardiac changes, including the autonomic system, could interfere with the control of different cognitive domains. Therefore, we assess the relationship between long-term heart rate variability (HRV), as a measure of autonomic nervous system (ANS) functioning, and cognitive performance in elderly patients representative of outpatients in a real-life setting.

**Methods**: Of 155 elderly outpatients (aged >65 years) screened, 117 enrolled patients underwent anthropometric evaluation, cardiac assessment by 12-lead electrocardiogram, 24-h ECG recording, and blood pressure (BP) measurement, as well as global cognitive evaluation by a standardized multidimensional assessment, including the Mini-Mental State Examination (MMSE) and Montreal Cognitive Assessment test (MoCA). HRV analysis was performed on 24-h ECG recordings focusing on time-domain indices [Standard deviation of the NN intervals (SDNN), standard deviations of 5-min mean values of the NN intervals for each 5-min interval (SDANN), and root mean squares of successive differences of the NN intervals (RMSSD)] and on frequency-domain measurements [heart rate (HR), low frequency (LF), high frequency (HF), and LF/HF]. Multivariate linear analysis was used to explore the influence of the HRV significant variables on MMSE and MoCA test values.

**Results**: The MMSE and MoCA scores were both significantly and positively correlated with the sympathetic system parameters (SDNN, SDANN, LF, and LF/HF ratio), but not with the parasympathetic system parameters (RMSSD and HF). Multivariate analysis confirms this relationship.

**Conclusions**: Our results show that, in a representative real-life community elderly population, an increased sympathetic activity, but not decreased vagal activity, is associated with better cognitive performances. These results support the sympathetic autonomic function, in that the relationship between better cognitive performances and a moderate prevalence of autonomic function appears dependent on long-term changes in heart rate, mediated by sympathetic activation.

## Introduction

Changes in cognitive performances and cardiovascular disorders represent a normal phenomenon of the aging process (Di Carlo et al., 2000; Leritz et al., 2011; Harada et al., 2013). Cardiovascular risk factors, such as smoking, obesity, diabetes mellitus, increased cholesterol, and systemic blood pressure (BP) levels, and an inadequate lifestyle may compromise also cerebral blood flow, which in turn can negatively affect cognitive performance (Takeda et al., 2017). Moreover, the same age-related anatomical and functional cardiac changes, including also the autonomic nervous system (ANS), determine cardiac output alteration, causing cerebral blood flow modulation. This variation could interfere with microcirculation and cause cerebral ischemia, particularly in those brain sites that control the different cognitive domains (Chen et al., 2011; Al-Qudah et al., 2015; Takeda et al., 2017). Past and recent evidence (Camm et al., 1996; Shaffer and Ginsberg, 2017; Singh et al., 2018a,b) support the relevance of ANS study by heart rate variability (HRV) assessment as a tool for the noninvasive analysis of cardiovascular autonomic function. HRV, defined as a marker identifying the balance between sympathetic and parasympathetic tone, predicts total mortality, sudden death, cardiovascular disease risk, as well as other morbidities (Sessa et al., 2018). It represents the measure of physiological variation in the interval between consecutive heart sinus beat or in the fluctuations between instantaneous heart rates and provides the importance that the ANS has regarding cardiovascular health and prognosis (Shaffer and Ginsberg, 2017). The parameters describing different HRV characteristics are generated by several algorithms ranging from simple mathematical or geometrical indices to complex nonlinear parameters. However, the interpretation of HRV parameters is complex and, in some cases, controversial (Camm et al., 1996). HRV, certainly, represents the evidence of the control of the ANS on the heart, but at the same time, HRV is very often used to describe the ANS functioning in general. Long-term HRV analysis (24 h) and short-term HRV analysis (5 min) are the two main methods for HRV analysis. The first is the time-domain assessment method, recorded over longer periods, traditionally 24 h, and the second is the frequency-domain assessment method, obtained by spectral analysis performed in shorter segments of 5 min as well as from the averaged over the entire 24-h period (Camm et al., 1996). In particular, a longer recording (24 h) better represents the daily activity, the circadian rhythms, and the response to environmental stimuli (Camm et al., 1996; Nunan et al., 2010; Kuusela, 2013; Sassi et al., 2015; Shaffer and Ginsberg, 2017; Singh et al., 2018a,b). On the contrary, short-term surveys (5 min) are suitable for outpatients, or to perform stimulation tests (Camm et al., 1996; Nunan et al., 2010; Kuusela, 2013; Sassi et al., 2015; Shaffer and Ginsberg, 2017; Singh et al., 2018a,b). Therefore, both methods are not interchangeable and should be used in a complementary way (Camm et al., 1996; Shaffer and Ginsberg, 2017; Singh et al., 2018a,b).

Although there are numerous studies in the literature evaluating the relationship between cognitive performance and cardiovascular risk factors, conversely, the relationship between ANS and cognitive function is less explored in the aged population (Britton et al., 2008; Nicolini et al., 2014). In particular, most previous studies tend to report different HRV measures by assessing them in different ways (during rest and/or during physical activities) and performing HRV in selected populations that do not always produce data consistent with common aging. The key issue is, therefore, the possibility of having effective data in contexts coherent with real life without compromising the effectiveness of the relative results.

Thus, the aim of the current study was to assess the relationship between HRV and cognitive performance in older patients representative of a real-life setting, during daily routine activities.

## Materials and Methods

### Subject Recruitment

**Figure 1:**
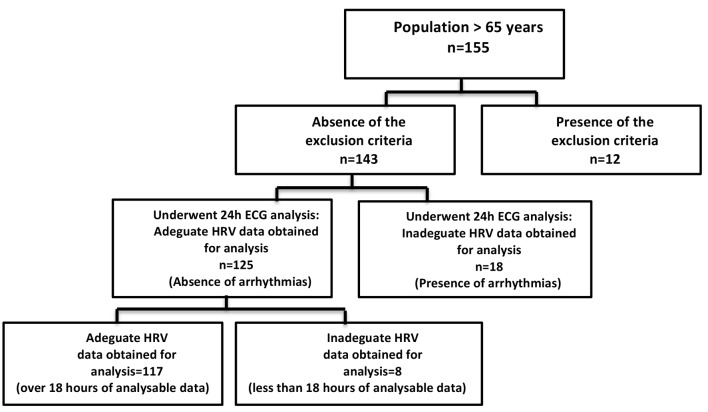
Flowchart of the study participants.

A population of 155 older subjects (aged >65 years) who, consecutively, underwent a standard geriatric visit at the outpatient Geriatric Centre of the University of Campania “Luigi Vanvitelli”—Naples, from January 2018 to December 2018, volunteered for the study (Figure 1). After a clear explanation of the study design, only 117 patients were enrolled, signing the informed consent form to participate in this study, which was approved by the Institutional Committee of ethical practice of our institution (approval number 630, Ethics Committee of the University of Campania L. Vanvitelli, Naples). The exclusion criteria were as follows:

(1) Health conditions with significant impact on HRV: competitive sports activity, clinical evidence of acute coronary artery disease in the last 3 months, significant valve disease, decompensated diabetes mellitus (HbA1c >8%), obesity [body mass index (BMI) >30 kg/m^2^], neurological and psychiatric diseases (degenerative and/or vascular dementia, Parkinson’s disease, stroke, and major depression diagnosis), and severe multi-organ diseases (anemia and/or pulmonary disease and/or cancer);
(2) Non-sinus rhythm (atrial fibrillation and other arrhythmias, paced rhythms) and the presence of numerous significant ventricular and supraventricular ectopic beats;
(3) Clinical signs and/or symptoms consistent with heart failure and evidence of left ventricular dysfunction (ejection fraction <50%);
(4) The use of psychotropic medications: tricyclic antidepressants, selective serotonin-noradrenaline reuptake inhibitors (SNRIs), atypical antidepressants, antipsychotics, and cholinesterase inhibitors; and
(5) Alcohol abuse or dependence in the last 2 years.

### Clinical Assessment

Anthropometric measurements (weight, height, and BMI), vital signs [BP and heart rate (HR)], and comorbidity (expressed as a percentage of the total cases studied) were evaluated. BP was recorded three times after 5 min of resting using a validated digital sphygmomanometer (GIMA spa, Italy). Socio-demographic data (age, gender, and education), lifestyle habits (mainly tobacco smoking, diet, and daily physical activity), and daily medication assumption were evaluated.

### Heart Rate and Heart Rate Variability Assessments

HR was performed by 12-lead ECG (EVO Esaote Delmar Reynolds), while 24 h of continuous ECG recordings were obtained using a Holter recorder (Spacelabs Healthcare EVO Digital software). Patients were asked to maintain a regular lifestyle and constant behavior during the 24-h Holter recording period. The 24-h ECG recordings started at about 10:00 a.m. and required a minimum of 18 h of analyzable data for the record to be accepted as valid. Artifacts and arrhythmia were visually detected and removed with the correction filters provided and incorporated from the EVO HRV Analysis software. The data were then exported to the HRV analysis software for analysis of the following HRV parameters: time-domain indices (SDNN, SDANN, and RMSSD) and frequency-domain measurements (HR, LF, HF, and LF/HF). In the time domain, the following parameters were calculated: standard deviation of the NN intervals (SDNN, in milliseconds), standard deviations of 5-min mean values of the NN intervals for each 5-min interval (SDANN, in milliseconds), and the root mean squares of successive differences of NN intervals (RMSSD, in milliseconds). In particular, SDNN reflects both longer-term circadian differences and total HRV, including both high-frequency variations and lower-frequency components observed over a 24-h period (Camm et al., 1996; Shaffer and Ginsberg, 2017). SDANN reflects the combined sympathetic and vagal activity. RMSSD reflects the cardiac parasympathetic activity. Lower SDNN values are associated with cardiovascular risk, higher SDANN values are considered beneficial, and higher RMSSD values reflect parasympathetic influencing (Camm et al., 1996; Shaffer and Ginsberg, 2017).

In the frequency domain, the power spectra of the following frequency bands were calculated: low frequency (LF, 0.04–0.15 Hz), high frequency (HF, 0.15–0.40 Hz), and the LF/HF ratio, expressed in normalized units (nu). LFn, HFn, and the LF/HF ratio represent an index of sympathetic modulation, an index of parasympathetic modulation, and an index of sympathovagal balance, respectively (Camm et al., 1996; Shaffer and Ginsberg, 2017). In 24-h recordings, the LF band power makes a significant contribution to SDNN (Camm et al., 1996; Shaffer and Ginsberg, 2017).

### Comprehensive Geriatric Assessment

#### Psychometric Evaluation

Global cognitive function was performed using standardized multidimensional assessments, including: (a) the Mini-Mental State Examination (MMSE; corrected for age and education) to provide an estimate of global cognitive functioning (Folstein et al., 1975); and (b) the Montreal Cognitive Assessment test (MoCA) to assess different cognitive domains (attention and concentration, executive functions, memory, language, constructive abstraction, calculation, and orientation; Nasreddine et al., 2005). The time of administration of both tests was 10–15 min. The score range is 0–30; higher scores indicate better cognitive function. MMSE scores ranging from 24 to 30 and MoCA scores ranging from 18 to 30 are considered within the normal range (Mitchell, 2009; Santangelo et al., 2015; Trzepacz et al., 2015). In several studies, the MMSE cutoff value of ≥24 or a score of ≥26 and ≥23 was also used (Christa Maree Stephan et al., 2013). Depressive symptoms by the Geriatric Depression Scale short version (GDS short version) were also investigated (score range 0–15, higher scores indicating greater depressive symptoms; Sheikh and Yesavage, 1986).

##### Functional Assessment

Activity functions were assessed by Basal Activities of Daily Living scale (BADL; Katz, 1963) and by Instrumental Activities of Daily Living (IADL; Lawton and Brody, 1969).

#### Statistical Analyses

Statistical analysis was performed using SPSS software packages (version 23). *A priori* power analysis was conducted before the study (G*power), which estimated that the minimal sample size needed was 84 participants (effect size = 0.5, err prob = 0.05, power 1 − *b* err prob = 0.95).

Normally distributed variables were described as the mean ± SD; non-normally distributed variables were described as medians and percentiles. A value of *p* < 0.05 was considered statistically significant. Correlation analysis was performed using Pearson’s correlation coefficient. Multivariate linear regression analysis, adjusted for all covariates, was used to explore the association of the autonomic nervous parameters and the degree of cognitive performance, evaluated by the MMSE and MoCA test. The collinearity of variables used in the statistical analyses was examined by calculating the variance inflation factor (VIF) using linear regression analysis. We accepted a maximum VIF of 10 to exclude multicollinearity problems.

## Results

According to the exclusion criteria, 117 (61 males and 56 females) of the 155 patients recruited for the study were suitable for analysis. Table 1 shows the baseline characteristics of the participants. All patients were aged, slightly overweight, on adequate glycemic and metabolic control, and had an education level mean of 6.2 years (range, 1–18 years). Only 10% of the study participants consume approximately five to eight cigarettes/day. Given the age, the ejection fraction of the left ventricle (LVEF) was found to be normal (53.1 ± 2.1%).

**Table 1:** Descriptive characteristics of the study group.

Characteristics	All groups = 117
Sex (M/F)	61/56
Age (years)	73.5 ± 6.9
BMI (kg/m^2^)	26.5 ± 3.8
FPG (mg/dl)	95 ± 7
Cholesterol (mg/dl)	174 ± 19
Triglycerides (mg/dl)	132 ± 27
HbA1c (%)	6.3 ± 0.89
HR from basal ECG (bpm)	71.8 ± 11.5
SBP (mmHg)	130 ± 15
DBP (mmHg)	77 ± 7
LVEF (%)	53.1 ± 2.1
Education (years)	6.2 ± 3.8

*Values are the mean ± SD. BMI, body mass index; FPG, fasting plasma glucose; HR, heart rate; SBP, systolic blood pressure; DBP, diastolic blood pressure; LVEF, left ventricle ejection fraction*.

Time and frequency variability values obtained from the 24-h Holter recordings and expressed as median are reported in Table 2. Patients showed longer NN intervals, signifying a prevailing sympathetic functioning. With regard to the frequency-domain measures, LF power was higher than HF power and the LF/HF ratio was resulted >1, thus showing a slight prevalence of the sympathetic system and suggesting a decreased parasympathetic control of heart rate (Table 2). Furthermore, all time-domain measures were higher in males compared to female patients.

**Table 2:** HRV parameters of the study group.

Parameters	Values	
HR max from 24 h-ECG (bpm)	99.6 ± 12.7
HR mean from 24 h-ECG (bpm)	69.8 ± 9.1	
HR min from 24 h-ECG (bpm)	55.2 ± 8.4
Markers of sympathetic function		
SDNN (ms)	104 ± 32.1	(83.6–123.8)
SDANN (ms)	92.7 ± 28.6	(77.2–107.5)
LF (nu)	47.5 ± 10.9	(39.7–54.5)
Markers of parasympathetic function		
RMSSD (ms)	32.3 ± 20.5	(23.6–45)
HF (nu)	41.3 ± 9.2	(36.3–46.5)
Markers of sympathovagal balance		
LF/HF	1.24 ± 0.7	(0.9–1.5)

*Values are the median ± SD (25th-75th percentile). HR, heart rate; bpm, beats per minutes; SDNN, standard deviation of all normal to normal intervals; SDANN, standard deviation of average normal to normal intervals; RMSSD, square root of the mean of the sum of the squares of differences between adjacent normal to normal intervals; LF, low frequency; HF, high frequency*.

By comparing the HRV parameters of males and females, we found significant differences for SDNN and SDANN (117.1 ± 36.7 vs. 97.3 ± 22.4, *p* < 0.001, and 103.9 ± 32.3 vs. 89.2 ± 21.9, *p* < 0.005, respectively), signifying an increased sympathetic functioning in male subjects, although males had the HR mean (70 ± 13 vs. 73 ± 9, *p* < 0.164) and HR mean from 24-h ECG (68 ± 9 vs. 70 ± 8, *p* < 0.303) lower than the females, perhaps because they were taking *β*-blockers.

Regarding global cognitive assessment (Table 3), the MMSE and MoCA scores show a mild cognitive decline. All cognition data were adjusted for the average number of educational years (6.2 ± 3.8 years). In particular, 69 (58.9%) and 81 (69.3%) patients show normal cognitive performances, while 48 (41.1%) and 36 (30.7%) patients show a mild cognitive decline, as revealed by both the MMSE and MoCA test, respectively. No significant cognitive profile differences were observed between males and females (Table 4).

**Table 3:** Cognitive assessment parameters of the study group.

Parameters	Values
MMSE (30 items)	23.3 ± 5.1
MoCA (30 items)	18.9 ± 5.6
GDS (15 items)	4.7 ± 2.9
ADL (6 items)	5.0 ± 0.8
IADL (8 items)	5.8 ± 2.2

*Values are the mean ± SD. MMSE, Mini-Mental State Examination; MoCA, Montreal Cognitive Assessment; GDS, Geriatric Depression Scale; ADL, activity of daily living; IADL, instrumental activity of daily living*.

**Table 4:** MMSE and MoCA test scores according to gender.

	Males	Females	*p*-value
MMSE	24.1 ± 4.9	22.4 ± 5.2	0.067
MoCA	19.9 ± 5.6	17.9 ± 5.5	0.053

*Values are the mean ± SD. MMSE, Mini-Mental State Examination; MoCA, Montreal Cognitive Assessment*.

The most frequently reported comorbidities included hypertension (54.9%), chronic kidney disease (28.4%), and type 2 diabetes mellitus (28.3%), all on adequate therapeutic control. Almost one-fifth of the patients (23.4%) were treated with *β*-blockers.

### Relationship Between Global Cognitive Function and HRV

The MMSE and MoCA scores were both significantly and positively correlated with the sympathetic system parameters (SDNN, SDANN, LF, and the LF/HF ratio), but not with the parasympathetic system parameters (RMSSD and HF; Table 5). No correlation was found between the MMSE and MoCA scores and LVEF (*r* = 0.861, *p* < 0.163, and *r* = 0.223, *p* < 0.173, respectively). Furthermore, the association between HRV and global cognitive function was also tested in multiple linear regression models (Table 6). The model including LF, HF, LF/HF ratio, SDNN, SDANN, and RMSSD as independent variables explained 40% and 43% of the MMSE and MoCA score variance, respectively. In such a multivariate model with MMSE as the dependent variable, the LF/HF ratio, in addition to BMI (*β* = 0.422, *p* = 0.001), represents the main determinant of MMSE score variability, while in a multivariate model with MoCA as the dependent variable, the SDNN variable, in addition to BMI (*β* = 0.223, *p* = 0.01) and the LF/HF ratio (*β* = 0.613, *p* = 0.001), represents the main determinant of MoCA score variability (Table 6). Given the possible high level of correlations between the HRV parameters, the multivariate model was inspected for multicollinearity by calculating the VIF, and no significant collinearity was found. In effect, the VIF was <10, thus confirming that the level of multicollinearity in this multivariate model is acceptable (Table 6).

**Table 5:** Correlation between the HRV parameters and the MMSE and MoCA test.

	MMSE	MoCA
SDNN (ms)	0.390**	0.481**
SDANN (ms)	0.324*	0.404**
RMSSD (ms)	0.156	0.099
LF (nu)	0.293**	0.336**
HF (nu)	−0.132	−0.135
LF/HF	0.248**	0.326**

SDNN, standard deviation of all normal to normal intervals; SDANN, standard deviation of average normal to normal intervals; RMSSD, square root of the mean of the sum of the squares of differences between adjacent normal to normal intervals; LF, low frequency; HF, high frequency. *p < 0.05; **p < 0.001

**Table 6:** Adjusted associations between the cognitive parameters and the individual components of HRV^a^.

	MMSE					MoCA					
	*B*	SEM	β	*t*	*p*-value	*B*	SEM	β	*t*	*p*-value	VIF
LF	−0.095	0.089	−0.202	−1.073	0.286	−0.144	0.095	−0.280	−1.519	0.132	5.195
HF	−0.028	0.053	−0.049	−0.524	0.602	0.016	0.056	0.026	0.287	0.775	1.534
LF/HF	**3.238**	**1.452**	**0.416**	**2.230**	**0.028**	**5.213**	**1.553**	**0.613**	**3.357**	**0.001**	5.072
SDNN	0.030	0.024	0.187	1.260	0.211	**0.068**	**0.026**	**0.387**	**2.660**	**0.009**	3.856
SDANN	0.029	0.028	0.162	1.027	0.307	0.033	0.030	0.165	1.070	0.287	4.343
RMSSD	0.023	0.021	0.092	1.127	0.263	−0.001	0.022	−0.005	−0.066	0.948	1.154

*LF, low frequency; HF, high frequency; SDNN, standard deviation of all normal to normal intervals; SDANN, standard deviation of taverage normal to normal intervals; RMSSD, square root of the mean of the sum of the squares of differences between adjacent normal to normal intervals; VIF, variance inflation factor. ^a^Based on linear regression analyses adjusted for age, sex, BMI, cigarette smoking, and β-blockers. The values shown in bold represent the statistically significant values (*p* < 0.05)*.

## Discussion

The aim of our study was to assess the association between HRV, as a measure of ANS functioning, and cognitive performance in elderly patients during daily activity, thus representing a real-life setting of the older population. The results suggest a connection between HRV and global cognitive performance, with a stronger association between the sympathetic parameters and better cognitive performance. In contrast, no correlation between the indices of parasympathetic modulation and cognitive performance was found. Our findings suggest that only increased sympathetic functioning, but not reduced parasympathetic modulation, implies a good contribution of sympathetic autonomic regulation to the overall cognitive function. Activation of the autonomic system has been reported to occur during cognitive assessment (Britton et al., 2008; Nicolini et al., 2014; Luque-Casado et al., 2016). Neuroimaging studies have already shown prefrontal cortex activation during cognitive functioning and autonomic system activation (Lane et al., 2009). Other studies have also evaluated ANS modulation using a combination of cardiovascular reflex tests in patients with Alzheimer’s disease (AD) and/or mild cognitive impairment (MCI), but contrasting data were reported. Depressed (de Vilhena Toledo and Junqueira, 2008), increased (Algotsson et al., 1995), and unchanged (Struhal et al., 2016) parasympathetic functions and increased sympathetic functions (de Vilhena Toledo and Junqueira, 2008) were observed. Moreover, only a few studies investigated autonomic function using the HRV. Zulli et al. (2005) evaluated the effect of HRV in subjects affected by AD and MCI using 24-h ECG monitoring. This latest study showed that both the LF and HF power spectral mean values were significantly lower in patients with AD compared to patients with MCI. In a cross-sectional study performed in 5-min electrocardiographic recordings focusing on changes from baseline to stimulation of sympathovagal balance, Nicolini et al. (2014) showed a reduced autonomic function in MCI patients. Unfortunately, the authors did not demonstrate the feasible occurrence of autonomic dysfunction in basal condition. da Silva et al. (2018), using a meta-analysis approach, showed a greater degree of autonomic dysfunction in patients affected by dementia. In contrast, lower levels of resting parasympathetic function have been related to poorer attention and executive function tasks during both stressful and non-stressful situations (Hansen et al., 2009). Unfortunately, there are some pitfalls in all the studies. First, multiple protocols used a meta-analysis approach, while others studied HRV by analyzing short-term measurements based on 5-min recordings, which does not correspond to a complete view of daily life. As reported by Shaffer and Ginsberg (2017), the 24-h HRV recordings achieve greater predictive power than short-term measurements. Therefore, our study design provided 24-h ECG recordings focusing on time-domain indices and on frequency-domain measurements. Although the biological interpretation of these indices is complex and debated, and many other mechanisms may influence these indices during long-term HRV analysis, our findings showed an increased sympathetic tone as expressed by high levels of LF, the LF/HF ratio, SDNN, and SDANN and as confirmed by the positive and significant correlation between the time-domain indices and the frequency-domain parameters with better cognitive performance. It is also true that the LF/HF ratio has long been considered as an index of sympathovagal balance, but this viewpoint has also been strongly criticized (Billman, 2013) because the physiological bases are not clear (Laborde et al., 2017). However, our data showed both high levels of LF and the LF/HF ratio, but, in particular, showed the significant correlation between the MMSE and MoCA cognitive tests and SDNN, representing this latter parameter as the gold standard for a more accurate evaluation of total circadian HRV (Singh et al., 2018a).

Besides the structural and functional changes in the ANS, the age-related brain functional declines also involve changes in receptor numbers and function. In fact, the number of intracerebral nicotinic receptors remarkably decreased in the elderly (Billman, 2013). This reduction can be compounded by the degeneration of cholinergic neurons in the basal forebrain, as occurs in AD (Gannon et al., 2015).

So far, the involvement of the cholinergic system in learning and memory processes is controversial. A possible role of receptors of the locus coeruleus (LC) in the pathogenesis of AD is supported by preclinical and clinical data showing that LC degeneration plays a significant role in AD pathogenesis (Mravec et al., 2014). The use of *β*-blockers also seems to support such hypothesis. The *β*-adrenergic receptors are involved in the enhancement of memory after emotional experiences (Gliebus and Lippa, 2007), and adrenergic signaling plays a role in the retrieval of intermediate-term contextual and spatial memories because the hippocampus receives dense input from adrenergic terminals (Ross et al., 2015). We cannot exclude that our data may be influenced by the use of *β*-blockers. However, our results were independent of the use of *β*-blockers, although used by patients in therapy for a long time, in turn producing pharmacological control of the disease itself.

Although females and males did not differ in numbers, our results show that males have better cognitive performance than females. Furthermore, according to gender, significant differences in the frequency-domain indices and in the analysis of the time-domain parameters were found. Recent studies showed gender influences on HRV indices (Voss et al., 2015). The mechanisms underlying the gender differences in autonomic function are not clear. The difference could be due to a decline in autonomic balance related to the expected decrement in estrogen (Voss et al., 2015), suggesting that, at least partly, hormonal factors play a role. This result is consistent with our data, which found higher SDNN and LF in males than in females.

Obesity is another aspect that needs to be emphasized. Indeed, it is known that obesity, cardiovascular disease, and diabetes have a detrimental effect on cognitive functioning (Yaffe et al., 2004; Whitmer et al., 2005). Moreover, the association between obesity and HRV is not completely clear. Most studies in the literature have shown that obesity is associated with a reduced HRV, indicative of a poorer ANS function. However, many other studies have shown that fat with central distribution, assessed with the measurement of waist circumference (Licht et al., 2010), is strongly associated with HRV compared to the general adiposity detected with BMI measurement. Our results demonstrated that all patients participating in the study were slightly overweight, while no patient was obese. Also, a normal BMI or being slightly overweight was associated with better cognitive performance. These data are in agreement with literature data showing that a low BMI reflecting poor health conditions and a malnutrition status are associated with a lower MMSE score or a decline in cognitive functioning (Sanders et al., 2016). Moreover, adipose tissue secretes a number of hormones (leptin, adiponectin, resistin, and visfatin) and inflammatory markers (TNF-α and IL-6) having the control function on the sympathetic nervous system and immune system, directly stimulating the sympathetic nervous system in the hypothalamus and/or inducing a state of low-grade inflammation that, in turn, can also stimulate the sympathetic nervous system (Ouchi et al., 2011).

Although the specific role of hypertension on cognitive function is known, our data do not provide any evidence for a negative impact of BP on cognition. Observational studies suggest that hypertension causes a vascular injury effect and cerebrovascular damage (Iadecola et al., 2016). In addition, chronically elevated BP exacerbates AD, contributing to dementia. However, judicious treatment of hypertension seems justified to protect vascular health and, consequently, brain health (Iadecola et al., 2016). These findings are important because they increase the possibility that treatment of hypertension may also contribute to reducing the development or progression of AD (Gorelick et al., 2011). Indeed, our results could be justified by compensated BP levels, values that were not far from the targets suggested by the American College of Cardiology guidelines (Whelton et al., 2018), thus exerting a neutral effect on cognition.

Our study is strengthened by the rigorous assessment of cognitive function that included the use of two validated cognitive screening instruments. The MMSE has been the most widely used cognitive screening instrument, while the MoCA is a relatively new cognitive screening instrument (Nasreddine et al., 2005; Mitchell, 2009; Santangelo et al., 2015; Trzepacz et al., 2015). Conversely to MMSE, the MoCA may be more sensitive to the assessment of complexity in executive function (Saczynski et al., 2015). Our data show potential advantages of the MoCA compared to the MMSE both in the assessment of cognitive performance and in affirming a stronger correlation between better cognitive performance and activation of the sympathetic system.

## Conclusion

To the best of our knowledge, this is the first study investigating the association between long-term HRV analysis, as a measure of autonomic functioning, focusing on time-domain indices and global cognitive performance in a representative real-life community elderly population. In the current study, cognitive performance was significantly correlated with all the parameters representative of the sympathetic nervous system (SDNN, SDANN, and LF/HF ratio), but not with the parameters representative of the parasympathetic nervous system (HF and RMSSD). This finding suggests that long-term changes in heart rate, mediated by ANS, affect cognitive functioning mainly through the sympathetic system branch than the parasympathetic system branch. Thus, the evaluation of how sympathetic autonomic regulation was associated with the overall cognitive function in elderly patients would provide an opportunity to explain how the cognitive neuronal framework functions.

Finally, the present study has several limitations that must be considered. First, this is a cross-sectional study that does not allow for causal interpretation, and, second, this study includes a small sample size of participants. However, the findings need to be confirmed in larger and longitudinal studies.

## Data Availability Statement

The datasets for this manuscript are not publicly available due to agreements involved within informed consent from participants. Requests to access the datasets should be directed to MR, mariarosaria.rizzo@unicampania.it.

## Ethics Statement

The studies involving human participants were reviewed and approved by Ethics Committee of University of Campania Luigi Vanvitelli—Napoli. The patients/participants provided their written informed consent to participate in this study.

## Author Contributions

All authors’ specific areas of contributions are listed below. MB and MR: study concept, design, analysis and interpretation of data. AD, RP, RF, and MR: acquisition of data. AD and MR: drafting of the manuscript. MB, AG, and MR: critical revision of the manuscript for important intellectual content.

## Conflict of Interest

The authors declare that the research was conducted in the absence of any commercial or financial relationships that could be construed as a potential conflict of interest.
